# Topical editorials, papers and commentaries: next 10 years

**DOI:** 10.1192/bjo.2026.11048

**Published:** 2026-05-21

**Authors:** Aileen A. O’Brien, Kamaldeep Bhui, Richard Williams, Jill Stavert, Cornelius Katona, Kenneth R. Kaufman

**Affiliations:** Population Health Research Institute, City St George’s University of London, UK; Department of Psychiatry, University of Oxford, UK; Wadham College, University of Oxford, UK; East London NHS Foundation Trust, London, UK; Oxford Health NHS Foundation Trust, Oxford, UK; Welsh Institute for Health and Social Care, University of South Wales – Glyntaff Campus, UK; Centre for Mental Health Practice Policy and Law Research, Edinburgh Napier University, UK; Helen Bamber Foundation, London, UK; Division of Psychiatry, University College London, UK; Departments of Psychiatry, Neurology, and Anesthesiology, Robert Wood Johnson Medical School, Rutgers University, USA; Department of Psychological Medicine, Institute of Psychiatry, Psychology & Neuroscience, King’s College London, UK

**Keywords:** Ethics, health economics, transcultural psychiatry, burden of disease, evidence-based mental health

## Abstract

Editorials and commentaries have a central role in shaping debate, priorities and values within psychiatry. Reflecting on the first decade of *BJPsych Open*, we consider how topical writing both responds to and helps define emerging scientific, social, ethical and political challenges. Looking ahead, we suggest that global instability, technological change and pressures on academic freedom will increasingly shape psychiatric discourse, underscoring the importance of editorial independence, methodological rigour and openness to the airing of contested ideas in guiding the journal’s next decade.

**Figure:**
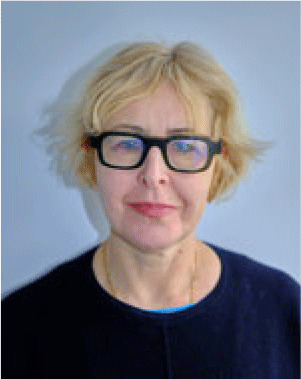

Editorials and expert commentaries give journals a distinctive voice and an influence over contemporary issues and may at times be contentious. As *BJPsych Open* enters its second decade, this editorial reflects on the contribution of topical writing to the journal’s first 10 years and considers the challenges and opportunities that are likely to shape its future direction. We first outline the distinctive function of editorials and commentaries within academic publishing, then review key themes that have characterised the journal’s output to date and finally identify emerging scientific, social, ethical and political issues that may define the next decade.

Rather than being ‘a collection of papers’,^1^ editorials provide an opportunity to reflect on specific topics and to influence research, clinical practice, teaching and the political determinants of poor health and societal opportunities. They can be the driver for evidence-based policy and for identifying priority research themes for future grant funding. These pieces can deepen understanding of complex topics, add nuance and present a particular perspective – potentially shaping professional and public opinion. Inevitably, they may sometimes be presented as provocative or polemic pieces to engage readers and policymakers despite their increasingly busy schedules and limited time. Therefore, it is important that editorials reflect relevant, topical and timely issues and that they are important and meaningful to the Journal’s stakeholders.^2^ What counts as ‘topical’ is shaped by many factors, both within psychiatry and in the wider world. The choice of subject matter must be significant and may be controversial. Against this backdrop, it is worth considering how *BJPsych Open* has enacted this role in practice.

Over its first decade, *BJPsych Open* has published more than one hundred editorials, topical papers and commentaries. They have covered many themes, addressing topics as varied as mental health stigma,^3^ refugee mental health,^4,5^ racism and riots,^6^ inflammation as a potential treatment target in mood disorders^7^ and caring for staff during and after the COVID-19 pandemic.^8^ Other issues that have seen traction in the wider literature include student mental health,^9^ social determinants of mental health,^10^ medical assistance in dying^11^ and treatment-resistant depression^12^ and psychosis.^13^ Which of these various areas will stand the test of time and which will turn out to reflect psychiatry’s susceptibility to succumbing to fleeting trends^14^ remains to be seen.

Editorials may link to research papers published in the journal, align with the interests of a particular group of authors, comment upon position statements of professional societies, or respond to current international events. Generally, they reflect a concern that something matters, something is at stake and that the Journal has both a duty and an opportunity to expose and articulate the concerns of constituencies and stakeholders in the spirit of promoting debate and informing progressive improvements in policy and practice.

Looking ahead, many global issues are likely to shape the journal’s focus. Money matters and global financial turbulence have had an inevitable impact on funding and delivering mental health services;^15^ health economics in psychiatry is likely to need to be an area of increasing focus and the Journal has a thematic series in progress on this topic. Artificial intelligence will shape every aspect of psychiatric practice and will change the lives of both staff and patients, driving scientific and clinical advances while also producing content and interventions whose impact we cannot yet fully predict.^16^

Global issues have an impact in shaping perceptions of priority for mental health provision, and related investments, alongside considerations of regional, national and cultural influences on the expression of mental distress and on what responsive care should look like. Examples of contentious issues include climate change and its mental health consequences, the growth of disaster psychiatry exposing geopolitical determinants of poor health and shifting support for Lower- and Middle-Income Countries which has recently seen disinvestment from global institutions. The needs of refugees have been and sadly will continue to be relevant given the influence of public attitudes to migration on the political life of all governments seeking election and retention of their political power (at least in the global north), the limited resources within which governments seek to care for their populations and the rising tides of far-right rhetoric and extremist anti-immigration attacks.

Stigma, social deprivation, armed conflicts, women’s mental health and future pandemics all demand attention. Women’s mental health requires a life-course approach, considering the impact of sex-based violence against women and girls, that of war, conflict and oppressive regimes, greater carer responsibilities and the medical neglect of pain, underdiagnosis of conditions, the impact of the perinatal period and menopause on mental and physical health and failure to recognise sex differences in pharmacological management.^17^

Young people’s and students’ mental health and well-being as well as barriers and access to care are increasingly relevant in times of global financial uncertainty. Legislative and ethical concerns such as changes to mental health law and the Medical Assistance in Dying for Mental Disorders debate are likely to have an impact on the day-to-day work of the readership and the lives of our patients.^18,19^ The increasing influence of human rights standards, particularly in relation to reducing psychiatric coercion, increasing autonomy and supporting a person’s wider needs^11^ are also central to law, policy and practice initiatives. It is crucial that we contribute to these national and international debates.

Academic publishing faces competing pressures, including market forces and the interests of powerful groups. The US attack on science and information makes scientific endeavour, ethical leadership and academic standards even more important. Maintaining editorial independence is vital. Journals like *BJPsych Open* must continue to promote robust academic discussion, even (indeed, especially) when it challenges prevailing views.

COPE, the Committee on Publication Ethics states that ‘undue influence by any political, corporate or social entity is against the core ethical principles of editorial independence and academic freedom’.^12^ We call upon all those involved in scholarly publishing to be guided by these principles when taking any action in response to external pressure.^13^ Central to maintaining the journal’s quality has been its focus on methodological rigour, research integrity and publication ethics, including attention to the ethical dimensions of clinical care.^2,13,14^ Academic freedom and its bedfellow of free speech are under pressure worldwide despite a lack of consensus on how these terms are interpreted, and measures are being introduced to protect free speech in higher education.^15^ The ability to step outside the hegemonic academic consensus within peer-reviewed journals is essential for avoiding ‘groupthink’ and for advancing psychiatry as a science. As Teixeira da Silva notes, science progresses through open exchange – by engaging with diverse ideas, even uncomfortable ones, and resisting pressures that might silence debate.^16^

The authors appreciate the significance of the topical editorials, commentaries and papers that have been published in *BJPsych Open* and the wider literature during the past decade. This commentary has provided an overview of selected publications and themes from the journal’s first decade and has identified emerging areas likely to shape future discourse. As editorial board members and clinical academics, we envision the expansion of such topical publications in the journal during the next decade and welcome submissions. Future priority topics should include, but are not limited to:

- global mental health;
- public mental health and prevention;
- social determinants of mental health;
- student mental health;
- sex, gender and mental health;
- diagnostic uncertainty and the problem of ‘overdiagnosis’;
- managing change, whether planned or emergent, and novel events and their impact on how people cope and
  support each other;
- artificial intelligence and digital mental health interventions;
- culturally adapted interventions and outcomes;
- novel therapeutics;
- biomarkers and genetics;
- treatment-resistant disorders;
- suicide and non-suicidal self injury;
- ethics in healthcare, research and publishing.
